# Relationship of urinary liver-type fatty acid-binding protein with cardiovascular risk factors in the Japanese population without chronic kidney disease: Sasayama study

**DOI:** 10.1186/s12882-021-02398-8

**Published:** 2021-05-21

**Authors:** Yoshimi Kubota, Aya Higashiyama, Mikio Marumo, Masami Konishi, Yoshiko Yamashita, Tomonori Okamura, Yoshihiro Miyamoto, Ichiro Wakabayashi

**Affiliations:** 1 Department of Environmental and Preventive Medicine, Hyogo College of Medicine, 1-1, Mukogawa-cho, Nishinomiya, Hyogo 663-8501 Japan; 2 Department of Hygiene, Wakayama Medical University, Wakayama, Japan; 3 Tamba-Sasayama City Office, Tamba-Sasayama, Hyogo Japan; 4 Department of Preventive Medicine and Public Health, School of Medicine, Keio University, Tokyo, Japan; 5 Open Innovation Center, National Cerebral and Cardiovascular Center, Suita, Osaka, Japan

**Keywords:** Liver-type fatty acid-binding protein, Albuminuria, Cohort studies, Diabetic kidney disease, Glomerular filtration rate, Japan

## Abstract

**Background:**

Urinary liver-type fatty acid-binding protein (L-FABP) is a well-known marker of proximal tubular impairment. We evaluated the relationship between cardiovascular disease (CVD) risk factors and levels of L-FABP in a cross-sectional community-based study. Participants with normoalbuminuria and normal estimated glomerular filtration rate (eGFR), that is, non-chronic kidney disease (non-CKD), were enrolled in this study. To the best of our knowledge, this is the first study to focus on the association between CVD risk factors and a proximal tubular marker in the Japanese general population with normoalbuminuria and normal eGFR.

**Methods:**

The present study is part of the Sasayama study. The participants included 1000 community residents (447 men and 553 women) aged 4064years without a history of CVD or renal dysfunction. Out of these participants 375 men and 477 women, defined as non-CKD, were included for further analysis. In each sex, the highest quintile group was considered to have high-normal L-FABP levels. A multiple logistic regression model was used to evaluate the relationship between risk factors for CVD and high-normal L-FABP levels in the non-CKD participants. We performed a similar analysis using the high-normal urinary albumin to creatinine ratio (UACR) as a dependent variable instead of L-FABP.

**Results:**

Among the non-CKD participants, in the highest quintile group (Q5, top 20%), L-FABP was 2.17g/gCre in men and2.83g/gCre in women. In women, the multivariate odds ratio was 3.62 (1.459.00) for high-normal L-FABP in the presence of diabetes mellitus (DM) compared with that in the group without DM. However, the relationship between DM and the UACR level was not significant. In men, DM was significantly associated with high-normal UACR. However, the relationship with L-FABP levels was not significant.

**Conclusions:**

The presence of DM was more strongly related to high-normal L-FABP levels than to high-normal UACR in women even at the stage of normoalbuminuria and normal eGFR. Our results were also consistent with the findings of a previous study where women were more prone to nonalbuminuric renal impairment compared to men, although further studies are required to confirm the results.

**Supplementary Information:**

The online version contains supplementary material available at 10.1186/s12882-021-02398-8.

## Background

Chronic kidney disease (CKD) not only progresses to end-stage renal disease (ESRD) but also represents an independent risk factor for cardiovascular disease (CVD) [1,3]. Early detection and prevention of CKD may thus contribute to extension of a healthy life expectancy. For diagnosis of CKD, estimated glomerular filtration rate (eGFR), urine total protein, and urine albumin are usually measured during medical check-ups or in clinical settings [4]. On the other hand, the early stages of renal impairment could be missed, as mentioned in previous studies [5,7]. A decline in the number of nephrons is accompanied by hypertrophy of the remaining nephrons. Although the prevalence of progressive eGFR decline is relatively low, a decline in renal function has been identified in follow-up studies not only in patients with microalbuminuria but also in patients with normoalbuminuria [5,7]. Results of in vitro and in vivo experiments, in previous studies, indicated that proximal tubular impairment preceded glomerular impairment in the kidneys of rats with diabetic nephropathy [8, 9]. We estimate that there is a considerable proportion of patients with early-stage renal impairment that is not detected by eGFR and urinary albumin. An easy-to-use marker for proximal tubules may facilitate diagnosis of these asymptomatic patients more definitively.

Urinary liver-type fatty-acid-binding protein (L-FABP) is expressed in the proximal tubules of the human kidney and plays a role in fatty acid metabolism [10]. It has been reported that expression of L-FABP in the proximal tubules is increased by ischemia or oxidative stress that causes tubulointerstitial damage [11]. Furthermore, urinary L-FABP has been used to predict deterioration of renal function in diabetic patients [11] and the occurrence of ESRD and CVD in patients with CKD [12]. It has previously been reported that risk factors of CVD such as hypertension, diabetes mellitus (DM), and dyslipidemia are associated with CKD [13, 14]. However, to date, there has been no study that focused on the association between CVD risk factors and a proximal tubular marker at the stage of normoalbuminuria and normal eGFR among the general population. Therefore, in this study, we evaluated the relationship between CVD risk factors and high-normal urinary L-FABP levels in Japanese residents with normoalbuminuria and normal eGFR.

## Methods

### Study population

**Fig. 1:**
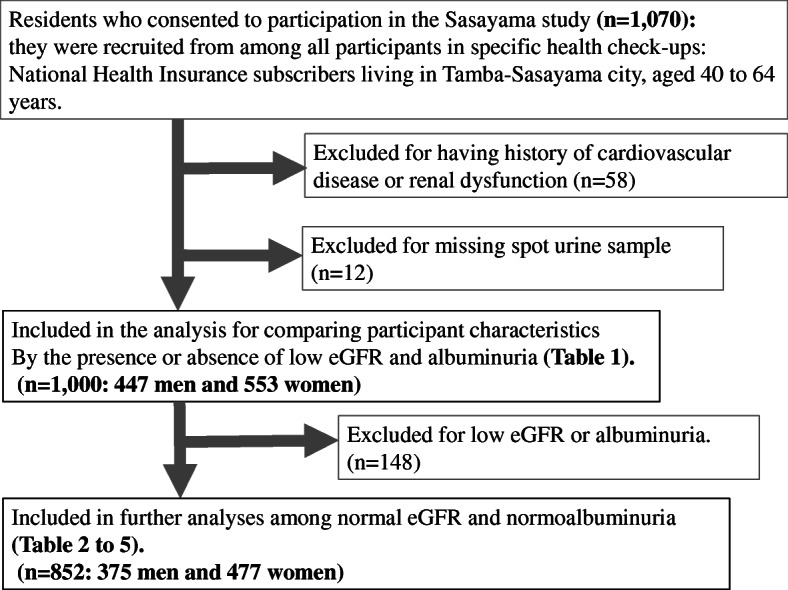
Flow chart of study population

The Sasayama study was a population-based cohort study, in which the assessed outcomes included, increased medical expenditures, worsening of quality of life, and CVD risk factors such as hypertension, DM, and dyslipidemia [15, 16]. The study cohort included healthy Japanese National Health Insurance subscribers (aged 40 to 64years) living in Tamba-Sasayama, a city in Hyogo prefecture, Japan, who underwent specific health check-ups. Since May 2012, we recruited participants in the Sasayama study from among all participants undergoing specific health check-ups who met the age and insurance type requirements. The annual consent rate for participation in the Sasayama study was approximately 77%. The present study was a cross-sectional study that used medical data recorded from May 2014 to February 2016. We excluded 70 participants for the following reasons: a history of cardiovascular disease or renal dysfunction (*n*=58), and missing spot urine sample (*n*=12). Consequently, the data of 1000 participants (447 men and 553 women) were included in the analysis. Whereby, the characteristics of participants, including the presence or absence of low eGFR and albuminuria, were compared. Of these participants, further analysis, using the data of 852 participants (375 men and 477 women) with normal eGFR (eGFR 60mL/min/1.73m^2^) and normoalbuminuria (UACR <30mg/gCre), was performed (Fig.1).

### Measurements

Single spot urine samples were collected and stored at 80C; urinary albumin, creatinine, and L-FABP were measured. Urinary L-FABP was measured by an enzyme-linked immunosorbent assay (ELISA) (CIMIC, Tokyo, Japan) [17]. The lower detection limit for the L-FABP assay was 1.5ng/mL and concentrations below the detection limit were defined as 0.75ng/mL using the mean value between zero and 1.5ng/mL. L-FABP levels were reported as ratios relative to urinary creatinine concentration (g/gCre). Urinary albumin concentration was measured by the turbidimetric immunoassay (TIA). Urinary albumin levels were also expressed as ratios relative to urinary creatinine (mg/gCre).

The eGFR was calculated using the following formula, which was specifically derived for the Japanese population: eGFR (mL/min/1.73m^2^)=194serum creatinine^1.094^age^-0.287^ (if female 0.739) [18]. In accordance with the Japanese Society of Nephrology guidelines [4], we defined eGFR <60mL/min/1.73m^2^ as low eGFR. We defined urinary albumin to creatinine (UACR)30mg/gCre as albuminuria then subdivided into UACR 30299mg/gCre as microalbuminuria and UACR 300mg/gCre as macroalbuminuria. Furthermore, participants showing eGFR <60mL/min/1.73m^2^ and/or urinary albumin 30mg/gCre were considered to have CKD. We also evaluated kidney impairment by using dipstick urinary protein scores using the same single urine sample immediately after sample provision; the participants showing scores of 1+ were considered to have proteinuria.

Blood samples were collected and serum creatinine, total cholesterol, high-density lipoprotein cholesterol (HDL-C), triglycerides, low-density lipoprotein cholesterol (LDL-C), and blood glucose were measured by enzymatic assays. The participants were considered to have hypertension if they had systolic blood pressure140mmHg and/or diastolic blood pressure90mmHg and/or had been prescribed medication for hypertension [19]; DM if they had fasting (for 10h) blood glucose levels 126mg/dL or non-fasting blood glucose levels 200mg/dL and/or Hb_A1c_6.5% and/or DM medication [20]; and dyslipidemia if they had LDL-cholesterol levels 140mg/dL and/or HDL-cholesterol <40mg/dL and/or non-HDL-cholesterol levels 170mg/dL and/or fasting triglyceride levels 150mg/dL and/or dyslipidaemia medication [21].

Smoking status, drinking habits, medication use, and medical history were assessed first during the medical check-up, according to the standard protocol for middle-aged and elderly individuals in Japan. Second, details of drinking habits were confirmed during face-to-face interviews with a research nurse and a nutritionist. Ethanol intake (g/day) was determined by alcohol consumption frequency, type of beverages consumed, and the amount of each type of beverage consumed per day. The participants with an ethanol intake of 46g per day, equivalent to approximately 2 g of the traditional Japanese unit of volume, were defined as heavy drinkers.

### Statistical analysis

The analyses were conducted according to the sex of the participants. Clinical characteristics of the participants with normal eGFR and normoalbuminuria were compared to those of the participants with albuminuria or low eGFR. Next, participants with normoalbuminuria and normal eGFR were included for further analysis, and were divided into quintiles by L-FABP (g/gCre) levels. As a biomarker of early kidney impairment before albuminuria and low eGFR occurrence, we defined high-normal L-FABP levels as the levels corresponding to the highest quintile (top 20%) group. .

Data are presented as meanstandard deviation (SD) for continuous variables and percentages for categorical variables. Variables with non-normal distribution are presented as medians and interquartile ranges (25, 75%). Bonferroni correction was applied to multiple post-hoc comparisons between continuous variables, and a Bonferroni-corrected chi-square test was used for categorical variables. For the non-CKD participants, multiple logistic regression analyses were performed to estimate the odds ratios (ORs) and the 95% confidence intervals (CIs) for high-normal L-FABP levels. Independent variables for multivariate analysis were traditional risk factors of CVD, including age, body mass index (BMI), hypertension, DM, dyslipidaemia, current smokers, and heavy drinkers. The UACR level was also included as an independent variable. To compare significant independent variables with high-normal L-FABP levels, multivariate OR and 95% CI for high-normal UACR were also estimated using the same CVD risk factors as independent variables. We divided the non-CKD participants according to UACR levels into quintiles and considered the highest quintile level (Q5) as high-normal UACR.

A two-sided *p*<0.05 was considered significant. The analyses were performed using IBM SPSS Statistics Version 22 for Windows (IBM, Tokyo, Japan).

## Results

The percentage of participants with and below the detection limit of L-FABP ( 1.5ng/mL) was 42.3% men and 56.2% women. When a value of 0.75ng/mL was assigned to the values lower than the detection limit and corrected by urinary creatinine, the median values of L-FABP were 1.33g/gCre in men and 1.71g/gCre in women. The percentage of participants showing L-FABP values that were equal to or higher than 8.4g/gCre (the normal upper limit for L-FABP [22]) were 1.3% men and 0.4% women. The levels of L-FABP in the highest quintile group (Q5, top 20%) were2.17g/gCre in men and2.83g/gCre in women among the participants with normal eGFR and normoalbuminuria (non-CKD).

The respective characteristics of participants with normal eGFR and normoalbuminuria, and with albuminuria and normal eGFR, and those with low eGFR are presented in Table1. In the groups of normal eGFR and albuminuria, 94.4% men and 90.9% women were considered to have microalbuminuria. In the groups of low eGFR, 98.1% men and 96.9% women had eGFR levels from 45 to 59mL/min/1.73m^2^. In men, L-FABP levels were significantly higher in the groups with normal eGFR and albuminuria than in other groups, while there was no significant difference in women. In both men and women, the group with normal eGFR and albuminuria had the highest levels of variables regarding DM (blood glucose and Hb_A1c;_ and the proportion of DM medication users).

**Table 1:**
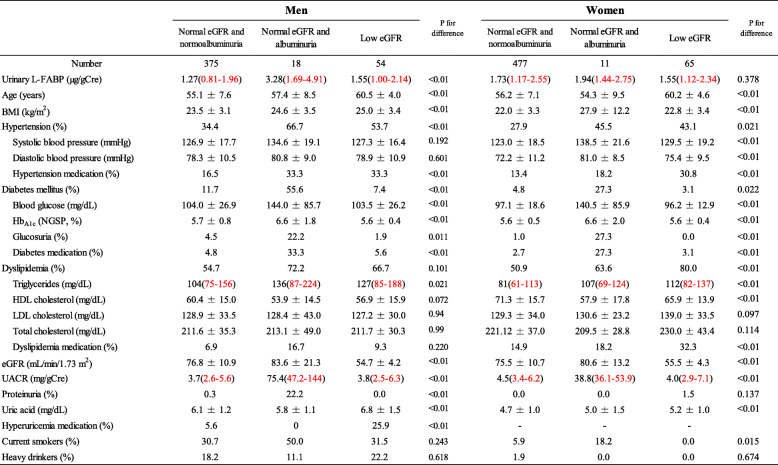
Participant characteristics by the presence or absence of low eGFR and albuminuria

Median and interquartile range (2575%)

Hypertension: systolic blood pressure140mmHg and/or diastolic blood pressure90mmHg and/or prescription of medication for hypertension

Diabetes mellitus: fasting blood glucose levels 126mg/dL or non-fasting blood glucose levels 200mg/dL and/or Hb_A1c_6.5% and/or prescription of medication for diabetes mellitus

Dyslipidemia: LDL-C levels 140mg/dL and/or HDL-C levels <40mg/dL and/or non-HDL cholesterol levels 170mg/dL and/or fasting triglyceride levels 150mg/dL and/or prescription of medication for dyslipidemia

*UACR* Urinary albumine to creatinine ratio (mg/gCre). Albuminuria: UACR 30mg/gCre. Low eGFR <60mL/min/1.73m^2^. Heavy drinker: 46g ethanol/day

In addition, participants with normoalbuminuria and normal eGFR were included for further analysis, and the participants characteristics are presented according to quintile L-FABP levels in Table2. Among the non-CKD participants, both in men and in women, the L-FABP levels were positively associated with age and UACR. In men, the L-FABP levels were positively associated with systolic blood pressure and inversely associated with HDL-cholesterol levels. In women, L-FABP levels were positively associated with the proportion of participants with DM and that of dyslipidaemia.

**Table 2:**
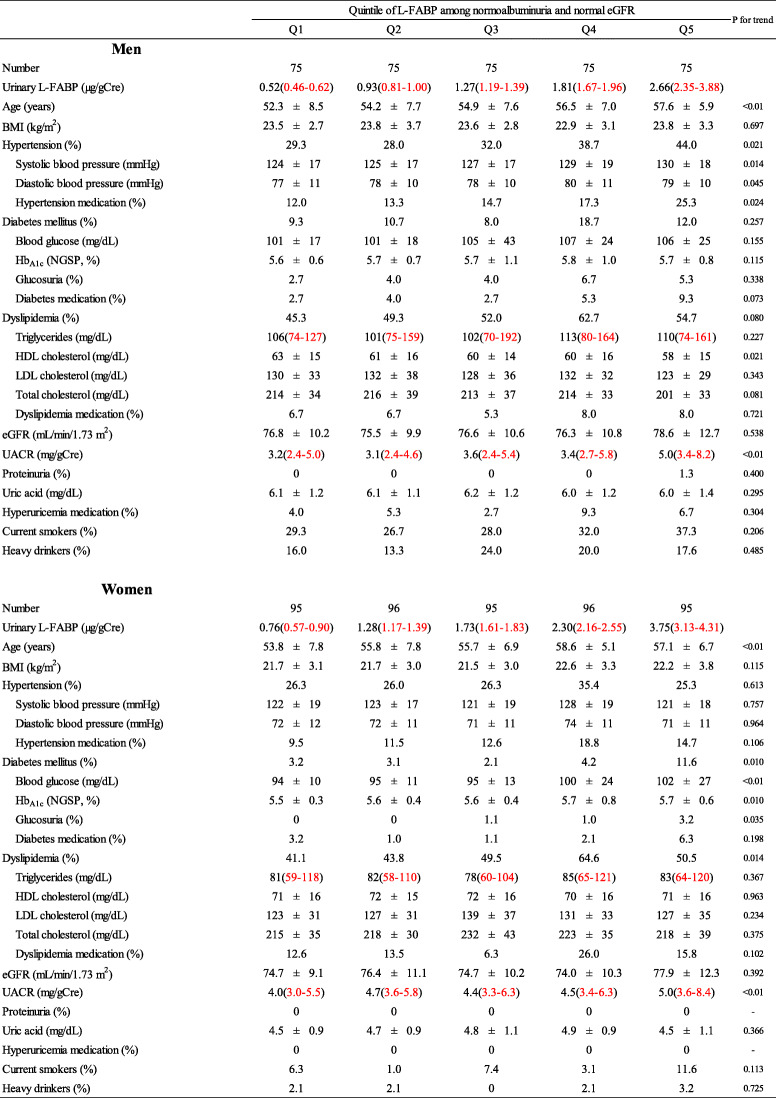
Participant characteristics according to quintile of L-FABP among non-CKD participants

Non-CKD: normoalbuminuria and normal eGFR

Median and interquartile range (2575%)

Hypertension: systolic blood pressure140mmHg and/or diastolic blood pressure90mmHg and/or prescription of medication for hypertension. Diabetes mellitus: fasting blood glucose levels 126mg/dL or non-fasting blood glucose levels 200mg/dL and/or Hb_A1c_6.5% and/or prescription of medication for diabetes mellitus. Dyslipidemia: LDL-C levels 140mg/dL and/or HDL-C levels <40mg/dL and/or non-HDL cholesterol levels 170mg/dL and/or fasting triglyceride levels 150mg/dL and/or prescription of medication for dyslipidemia

UACR: urinary albumine to creatinine ratio (mg/gCre). Albuminuria: UACR 30mg/gCre. Low eGFR <60mL/min/1.73m^2^. Heavy drinker: 46g ethanol/day

We also examined the characteristics of participants with non-CKD, according to quintile UACR levels (Table3). In both men and women, UACR levels were positively associated with age and the proportion of participants with hypertension. In men, UACR levels were positively associated with blood glucose, Hb_A1c_ and the proportion of participants with DM. We observed a similar relationship regarding Hb_A1c_ in women. However, the relationships were not significant for blood glucose and the proportion of participants with DM.

**Table 3:**
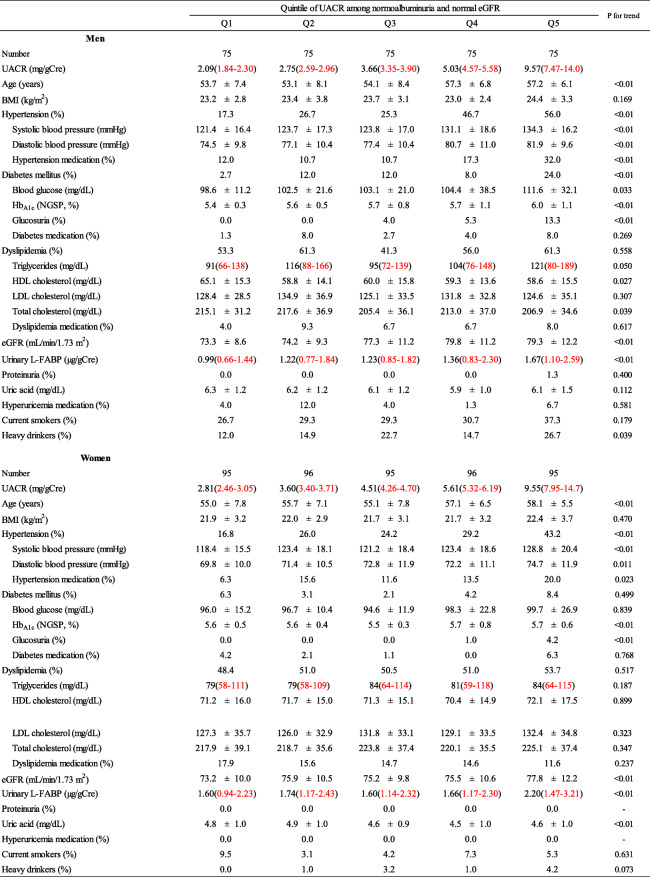
Participant characteristics according to quintile of UACR among non-CKD participants

Non-CKD: normoalbuminuria and normal eGFR

Median and interquartile range (2575%)

Hypertension: systolic blood pressure140mmHg and/or diastolic blood pressure90mmHg and/or prescription of medication for hypertension. Diabetes mellitus: fasting blood glucose levels 126mg/dL or non-fasting blood glucose levels 200mg/dL and/or Hb_A1c_6.5% and/or prescription of medication for diabetes mellitus. Dyslipidemia: LDL-C levels 140mg/dL and/or HDL-C levels <40mg/dL and/or non-HDL cholesterol levels 170mg/dL and/or fasting triglyceride levels 150mg/dL and/or prescription of medication for dyslipidemia

UACR: urinary albumine to creatinine ratio (mg/gCre). Albuminuria: UACR 30mg/gCre. Low eGFR <60mL/min/1.73m^2^. Heavy drinker: 46g ethanol/day

Table4 shows the associations between CVD risk factors and high-normal L-FABP levels (Q5) in the participants with normoalbuminuria and normal eGFR (non-CKD) by multiple logistic regression analysis. First, we calculated the crude OR (95%CI) for each independent variable as univariate analysis. Second, we examined three multivariate models using different sets of independent variables. When UACR levels (log-transformed) were included within the independent variables, in men, age was significantly associated with high-normal L-FABP [OR (95%CI): 1.07 (1.021.11)]. In women, the OR (95%CI) of presence of DM was 3.62 (1.459.00) and that of current smoking was 3.21 (1.317.85). Both in men and in women, the UACR levels were associated with high-normal L-FABP levels; the ORs were 2.56 (1.644.00) in men and 2.12 (1.373.27) in women. Following multivariate analysis, with an adjustment for age, BMI, systolic blood pressure, blood glucose, HDL-cholesterol (as continuous variables), smoking status (current smoking or not) and drinking status (heavy drinker or not), blood glucose levels and current smoking were associated with high-normal L-FABP levels in women; the OR of blood glucose levels was 1.01 (1.001.02) and that of current smoking was 2.90 (1.197.08). In men, age was associated with high-normal L-FABP levels; OR, 1.07 (1.031.12) (Supplement Table1).

**Table 4:**
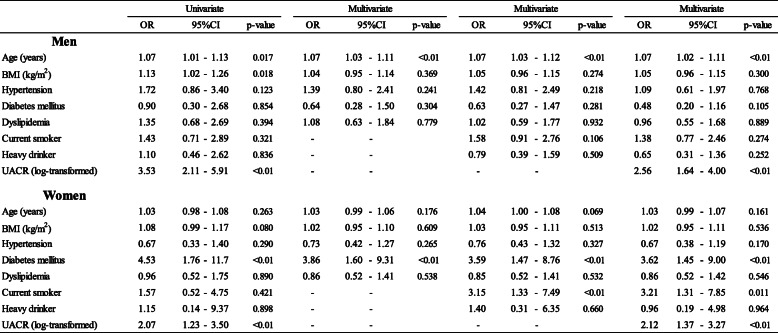
OR of high-normal L-FABP levels (Q5) associated with CVD risk factors among non-CKD participants

Non-CKD: normoalbuminuria and normal eGFR

Hypertension: systolic blood pressure140mmHg and/or diastolic blood pressure90mmHg and/or prescription of medication for hypertension. Diabetes mellitus: fasting blood glucose levels 126mg/dL or non-fasting blood glucose levels 200mg/dL and/or Hb_A1c_6.5% and/or prescription of medication for diabetes mellitus. Dyslipidemia: LDL-C levels 140mg/dL and/or HDL-C levels <40mg/dL and/or non-HDL cholesterol levels 170mg/dL and/or fasting triglyceride levels 150mg/dL and/or prescription of medication for dyslipidemia

UACR: urinary albumine to creatinine ratio (mg/gCre). Albuminuria: UACR 30mg/gCre. Low eGFR <60mL/min/1.73m^2^. Heavy drinker: 46g ethanol/day

Table5 shows the associations between CVD risk factors and high-normal UACR levels (highest quintile: Q5) in the non-CKD participants by multiple logistic regression analysis. The highest quintile levels of UACR (<30mg/gCre) were6.423mg/gCre in men and6.90mg/gCre in women. When L-FABP levels (log-transformed) were included within the independent variables in men, the OR (95%CI) of DM was 3.06 (1.456.49). Both in men and in women, hypertension was associated with high-normal UACR; the ORs were 2.25 (1.273.99) in men and 2.11 (1.263.53) in women.

**Table 5:**
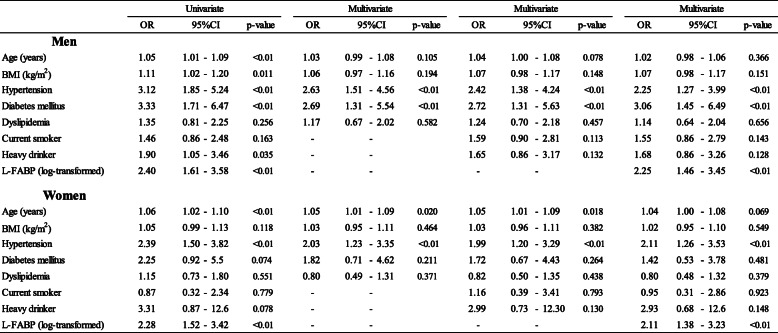
OR of high-normal UACR levels (Q5) associated with CVD risk factors among non-CKD participants

Non-CKD: normoalbuminuria and normal eGFR

Hypertension: systolic blood pressure140mmHg and/or diastolic blood pressure90mmHg and/or prescription of medication for hypertension. Diabetes mellitus: fasting blood glucose levels 126mg/dL or non-fasting blood glucose levels 200mg/dL and/or Hb_A1c_6.5% and/or prescription of medication for diabetes mellitus. Dyslipidemia: LDL-C levels 140mg/dL and/or HDL-C levels <40mg/dL and/or non-HDL cholesterol levels 170mg/dL and/or fasting triglyceride levels 150mg/dL and/or prescription of medication for dyslipidemia

UACR: urinary albumine to creatinine ratio (mg/gCre). Albuminuria: UACR 30mg/gCre. Low eGFR <60mL/min/1.73m^2^. Heavy drinker: 46g ethanol/day

## Discussion

We examined the association between CVD risk factors and high-normal L-FABP levels, which is a recently described marker of tubulointerstitial impairment among the general population with normoalbuminuria and normal eGFR. We found that hypertension was significantly associated with high-normal UACR levels in men and women. Of note, according to gender, DM was strongly associated with different renal impairment markers; high-normal UACR in men and high-normal L-FABP in women. Furthermore, DM and current smoking were significantly related to high-normal L-FABP levels in women only.

Using the dataset of participants without CKD, L-FABP levels were substantially lower in our present study than in a previous study [23]. In previous studies, L-FABP levels were measured for patients with DM or apparent renal failure [11, 23]. To the best of our knowledge, the present study is the first that investigated the relationship between L-FABP and CVD risk factors in normal eGFR and normoalbuminuria participants in a general population.

In a previous study, patients who failed to achieve and maintain blood pressure control during a 4-year follow-up showed an increased risk of developing diabetic nephropathy (OR 1.38, *p*<0.001) and risk of its component: albuminuria (OR 1.47, *p*<0.001) [24]. Penno et al. demonstrated that the female sex correlated with nonalbuminuric renal impairment and the male sex with the albuminuric forms in patients with type 2 DM [25]. A previous clinical study showed that urinary excretion of human L-FABP levels were correlated with the severity of tubuointertistial damage and the rate of CKD progression [11]. Structurally, L-FABP has a high capacity to bind and a high affinity for fatty acid peroxidation products and causes their excretion into urine and thus L-FABP may be an effective endogenous antioxidant [26]. Furthermore, Kanaguchi et al. evaluated the expression of tubulointerstitial markers under oxidative stress and proximal tubular damage in human L-FABP chromosomal gene-transgenic mice and suggested that tubular enhancement of L-FABP may protect mice with antibody-induced glomerulonephritis during the progression of both tubulointerstitial and glomerular injury [27]. Therefore, L-FABP is considered as a stress marker, which is increased by oxidative stress during ischemia of the proximal tubules and hyperglycaemia [11]. In addition, its potential protective effect against the progression of both tubulointerstitial and glomerular damage has been suggested, although further studies are required to confirm the detailed mechanisms [26, 27]. Neugarten et al. conducted a meta-analysis of 68 studies and concluded that men with CKD, due to various aetiologies, showed a more rapid time-dependent decline in renal function than did women [28]. In most experimental animal models of CKD, males showed a more accelerated progression of renal injury compared with females, and as a potential mechanism for renal protection in females, an influence of oestrogen is suggested [29]. In our study, in women, DM and current smoking, which cause oxidative stress, were associated with high-normal L-FABP levels that reflect oxidative stress in the proximal tubules. Additionally, considering the protective role of L-FABP against the progression of renal impairment, higher L-FABP levels in women compared to men may partly be explained by previous suggestions that the progression of renal impairment is slower in women than in men [28, 29]. Although there are few studies in which urinary L-FABP is measured separately according to gender, Ishimitsu et al. determined the average urinary L-FABP levels (g/g Cre) of 550 subjects without disease and L-FABP levels were significantly higher in women than in men, which is consistent with our findings [30].

In our study, L-FABP levels correlated strongly with UACR levels both in men and women, but particularly in men. It has been shown that tubular biomarkers, including L-FABP, were higher in patients with micro and macro-albuminuria compared to persons without albuminuria [9]. When either tubular or glomerular injuries occur, tubules and glomerulus closely interacted via glomerulotubular junctions [9, 31]. The stronger association between L-FABP and UACR levels in men rather than in women may partly contribute to a more rapid progression of renal injury in men.

Our study has several limitations. First, the determination of L-FABP levels and diagnosis of kidney impairment were based on data of participants in the research survey only. Second, the present study is a cross-sectional study and follow-up studies are needed to confirm that high-normal L-FABP levels, which reflect mild tubulointerstitial stress, predict glomerular impairment, and ultimately lead to a decline in kidney function. Third, the reason for the gender difference in the strength of the relationship between DM and high-normal L-FABP levels remains to be clarified.

## Conclusions

In conclusion, the presence of DM was more strongly related to high-normal L-FABP levels than high-normal UACR in women even at the stage of normoalbuminuria and normal eGFR, which reflects oxidative stress in the proximal tubules. Our results were also consistent with the findings of a previous study where women were more prone to nonalbuminuric renal impairment than men [25], although further studies are required to confirm the results.

## Supplementary Information

**Additional file 1: Supplement Table 1**. OR of high-normal L-FABP levels (Q5) associated with CVD risk factors among normoalbuminuria and normal eGFR participants.

## Data Availability

The datasets generated and/or analysed during the current study are not publicly available due to restrictions based on privacy regulations and informed consent of the participants, but are available from the corresponding author on reasonable request.

## Abbreviations

BMI: Body mass index
CVD: Cardiovascular disease
CKD: Chronic kidney disease
CI: Confidence interval
DM: Diabetes mellitus
eGFR: Estimated glomerular filtration rate
ESRD: End-stage renal disease
L-FABP: Liver-type fatty acid-binding protein
OR: Odd ratios
PTEC: Proximal tubule epithelial cells
UACR: Urinary albumin to creatinine ratio

## References

[1] Nitsch D, Grams M, Sang Y, Black C, Cirillo M, Djurdjev O (2013). Associations of estimated glomerular filtration rate and albuminuria with mortality and renal failure by sex: a meta-analysis. BMJ.

[2] Weiner DE, Tighiouart H, Amin MG, Stark PC, Mac Leod B, Griffith JL (2004). Chronic kidney disease as a risk factor for cardiovascular disease and all-cause mortality: a pooled analysis of community-based studies. J Am Soc Nephrol.

[3] Matsushita K, van der Velde M, Astor BC, Woodward M, Levey AS, Chronic Kidney Disease Prognosis Consortium (2010). Association of estimated glomerular filtration rate and albuminuria with all-cause and cardiovascular mortality in general population cohorts: a collaborative meta-analysis. Lancet.

[4] Japanese Society of Nephrology (2012). Clinical practice guidebook for diagnosis and treatment of chronic kidney disease.

[5] Mac Isaac RJ, Tsalamandris C, Panagiotopoulos S, Smith TJ, McNeil KJ, Jerums G (2004). Nonalbuminuric renal insufficiency in type 2 diabetes. Diabetes Care.

[6] Babazono T, Nyumura I, Toya K, Hayashi T, Ohta M, Suzuki K (2009). Higher levels of urinary albumin excretion within the normal range predict faster decline in glomerular filtration rate in diabetic patients. Diabetes Care.

[7] Perkins BA, Ficociello LH, Ostrander BE, Silva KH, Weinberg J, Warram JH (2007). Microalbuminuria and the risk for early progressive renal function decline in type 1 diabetes. J Am Soc Nephrol.

[8] Gilbert RE, Cooper ME (1999). The tubulointerstitium in progressive diabetic kidney disease: more than an aftermath of glomerular injury?. Kidney Int.

[9] Zeni L, Norden AGW, Cancarini G, Unwin RJ (2017). A more tubulocentric view of diabetic kidney disease. J Nephrol.

[10] Kamijo A, Sugaya T, Hikawa A, Yamanouchi M, Hirata Y, Ishimitsu T (2005). Clinical evaluation of urinary excretion of liver-type fatty acid-binding protein as a marker for the monitoring of chronic kidney disease: a multicenter trial. J Lab Clin Med.

[11] Kamijo-Ikemori A, Sugaya T, Ichikawa D, Hoshino S, Matsui K, Yokoyama T (2013). Urinary liver type fatty acid binding protein in diabetic nephropathy. Clin Chim Acta.

[12] Matsui K, Kamijo-Ikemori A, Imai N, Sugaya T, Yasuda T, Tatsunami S (2016). Clinical significance of urinary liver-type fatty acid-binding protein as a predictor of ESRD and CVD in patients with CKD. Clin Exp Nephrol.

[13] Iseki K, Asahi K, Moriyama T, Yamagata K, Tsuruya K, Yoshida H (2012). Risk factor profiles based on estimated glomerular filtration rate and dipstick proteinuria among participants of the specific health check and guidance system in Japan 2008. Clin Exp Nephrol.

[14] Sakurai M, Kobayashi J, Takeda Y, Nagasawa SY, Yamakawa J, Moriya J (2016). Sex differences in associations among obesity, metabolic abnormalities, and chronic kidney disease in Japanese men and women. J Epidemiol.

[15] Higashiyama A, Kubota Y, Marumo M, Konishi M, Yamashita Y, Nishimura K (2015). Association between serum long-chain n-3 and n-6 polyunsaturated fatty acid profiles and glomerular filtration rate assessed by serum creatinine and cystatin C levels in Japanese community-dwellers. J Epidemiol.

[16] Kubota Y, Higashiyama A, Marumo M, Konishi M, Yamashita Y, Tashiro C (2017). Detection of subclinical peripheral artery ischemia in healthy male smokers by an ankle-brachial index after exercise: Sasayama study. Angiology..

[17] Kamijo A, Kimura K, Sugaya T, Yamanouchi M, Hikawa A, Hirano N (2004). Urinary fatty acid-binding protein as a new clinical marker of the progression of chronic renal disease. J Lab Clin Med.

[18] Matsuo S, Imai E, Horio M, Yasuda Y, Tomita K, Nitta K (2009). Collaborators developing the Japanese equation for estimated GFR. Revised equations for estimated GFR from serum creatinine in Japan. Am J Kidney Dis.

[19] Guidelines for the management of hypertension. Japan Society of Hypertension. 2019.

[20] Japanese Clinical Practice Guideline for Diabetes. The Japan Diabetes Society. 2019.

[21] Japan Atherosclerosis Society (JAS) Guidelines for Prevention of Atherosclerotic Cardiovascular Diseases 2017. Japan Atherosclerosis Society 2017. 10.5551/jat.GL2017 PMC6143773 30135334

[22] Kamijo-Ikemori A, Sugaya T, Yasuda T, Kawata T, Ota A, Tatsunami S (2011). Clinical significance of urinary liver-type fatty acid-binding protein in diabetic nephropathy of type 2 diabetic patients. Diabetes Care.

[23] Suzuki K, Babazono T, Murata H, Iwamoto Y (2005). Clinical significance of urinary liver-type fatty acid-binding protein in patients with diabetic nephropathy. Diabetes Care.

[24] De Cosmo S, Viazzi F, Piscitelli P, Giorda C, Ceriello A, Genovese S (2016). AMD-annals study group. Blood pressure status and the incidence of diabetic kidney disease in patients with hypertension and type 2 diabetes. J Hypertens.

[25] Penno G, Solini A, Bonora E, Fondelli C, Orsi E, Zerbini G (2011). Renal insufficiency and cardiovascular events (RIACE) study group. Clinical significance of nonalbuminuric renal impairment in type 2 diabetes. J Hypertens.

[26] Xu Y, Xie Y, Shao X, Ni Z, Mou S (2015). L-FABP: a novel biomarker of kidney disease. Clin Chim Acta.

[27] Kanaguchi Y, Suzuki Y, Osaki K, Sugaya T, Horikoshi S, Tomino Y (2011). Protective effects of L-type fatty acid-binding protein (L-FABP) in proximal tubular cells against glomerular injury in anti-GBM antibody-mediated glomerulonephritis. Nephrol Dial Transplant.

[28] Neugarten J, Acharya A, Silbiger SR (2000). Effect of gender on the progression of nondiabetic renal disease: a meta-analysis. J Am Soc Nephrol.

[29] Kummer S, von Gersdorff G, Kemper MJ, Oh J (2012). The influence of gender and sexual hormones on incidence and outcome of chronic kidney disease. Pediatr Nephrol.

[30] Ishimitsu T, Ohta S, Saito M, Teranishi M, Inada H, Yoshii M (2005). Urinary excretion of liver fatty acid-binding protein in health-check participants. Clin Exp Nephrol.

[31] Chevalier RL (2016). The proximal tubule is the primary target of injury and progression of kidney disease: role of the glomerulotubular junction. Am J Physiol Ren Physiol.

